# Editorial: Education and learning for digital health

**DOI:** 10.3389/fdgth.2023.1165504

**Published:** 2023-03-24

**Authors:** Elizabeth Morrow, Fiona Ross, Cindy Mason

**Affiliations:** ^1^ Research Support Northern Ireland, Downpatrick, United Kingdom; ^2^ Faculty of Health, Science, Social Care and Education, Kingston University London, London, United Kingdom; ^3^ AI Researcher (Independent), Palo Alto, CA, United States

**Keywords:** digital health, health technology adoption and use, health professional education and training, virtual learning, virtual care

**Editorial on the Research Topic** Education and learning for digital health

At a time of rapid digital innovation, the Research Topic, *Education and Learning for Digital Health* sparks novel thinking and insights into how and why health professionals learn to use emerging technologies. The Research Topic builds on established educational practices and associated works of literature on e-learning, blended learning, immersive virtual reality, digital simulations, and virtual patients. See for example the Frontiers Research Topic, *Advancing Teaching and Learning in Health Sciences Across Healthcare Professionals*. Many robust educational resources and tools for digital health have been developed, including Health Education England's e-learning for health (https://www.e-lfh.org.uk/). The COVID-19 pandemic impelled further innovation in health professionals’ online learning as education providers worked tirelessly to avoid disruption to learning, as captured in the Frontiers Research Topic, *Impact of COVID-19 on Healthcare Professions Education*.

The articles in this Research Topic, which have been produced by *17 world-leading clinicians, researchers, and educators* from Australia, Canada, the United States, and the Republic of Ireland, together with the expertise of article editors and peer reviewers from Finland, Netherlands, Ireland, Australia, the United Kingdom, United States, and Canada, advance the scientific paradigm of the discipline in four specific areas:

• Health professionals learning to adapt and use virtual care (VC)
• Digital professionalism in the use of smartphone technologies
• Preparing medical students to use artificial intelligence (AI) and machine learning (ML)
• Safe use of virtual reality (VR) technologies in professional education

It is significant that the international collaborations, studies, and articles on this Research Topic were all produced during the pandemic. In effect, this context ignited digital health by necessity in order to maintain clinical care during social distancing and infection control protocols, while safeguarding human rights and preserving the ethics of healthcare.

For example, in the first article, authors Vernon Curran, Ann Hollett, and Emily Peddle explain how the use of virtual care, such as virtual examinations, clinical assessments, and remote patient monitoring, expanded during COVID-19 to enable continued access to healthcare. Their survey study, *Virtual Care and COVID-19: A Survey Study of Adoption, Satisfaction, and Continuing Education Preferences of Healthcare Providers in Newfoundland and Labrador, Canada*, provides insights into healthcare providers’ experiences during the unfolding pandemic. It demonstrates that not all VC methods were perceived to deliver the same quality of care that would be expected in traditional face-to-face clinical encounters, but there can be other advantages for maintaining virtual proximity to patients whilst minimising the risk of infection transfer. As a result, the authors recommend healthcare provider organisations ensure VC is backed up with Continuing Professional Development (CPD), guidelines, and resources including patient educational support.

The second article focuses on the legal and ethical dimensions of smartphone technology. From the Republic of Ireland, Bernadette John, Christine McCreary, and Anthony Roberts authored *Smartphone Technology for Clinical Communication in the COVID-19 Era: A Commentary on the Concerning Trends in Data Compliance*. They argue that smartphone technologies afforded clinicians and patients many observed advantages during COVID-19, yet the long-term use of such devices needs to be compliant with protecting patient data security and privacy. Solutions offered include healthcare institutional guidelines, supportive digital professionalism training, and education opportunities.

The authors of the third article in this Research Topic suggest that changes to support future healthcare should begin in medical schools. From the United States, authors Timothy Frommeyer, Reid Fursmidt, Michael Gilbert, and Ean Bett elaborate on *The Desire of Medical Students to Integrate Artificial Intelligence Into Medical Education: An Opinion Article*. They draw on their wealth of experience in precision medicine, drug discovery, diagnostics, and hospital administration to argue that the advancement of AI and machine learning algorithms are reshaping the way physicians and healthcare providers approach the practice of medicine. They call for medical schools across the world to take up their essential educational role to ensure that changes to healthcare are for the better and that future physicians will be more competent, inventive, and compassionate in the medicine of tomorrow.

The fourth article considers how, alongside changes in curricula content, advanced technologies are changing the modes of education delivery to provide digitally enhanced learning experiences. From Australia, authors Nathan Moore, Kathy Dempsey, Peter Hockey, Susan Jain, Philip Poronnik, Ramon Shaban, and Naseem Ahmadpour explain their work on *Innovation During a Pandemic: Developing a Guideline for Infection Prevention and Control to Support Education Through Virtual Reality*. Their article focuses on virtual reality as an educational technology with the ability to deliver flexible and immersive education. Their attention to safe infection control practices of VR head-worn display systems is ensuring safer transfer between clinicians.

The insights from these articles show that education and learning for digital health need to address a growing range of patient rights, professional practice, and governance issues. The issues extend from the level of individual practitioner's use of technologies protecting patient confidentiality; to institutional policies, data licencing, copyright agreements, and intellectual property rights; to whole health system design and regulation of digital health technologies; as well as raising public awareness and trust in such advances (1).

Looking to the future, it is through the combination of education and learning in humans and machines that new knowledge will gain the greatest power to maximise well-being, as explored in the related Frontiers Research Topic, *The Good Side of Technology: How We Can Harness the Positive Potential of Digital Technology to Maximize Well-being*. The design and use of advanced technologies in healthcare are increasingly looking beyond hybrid and “human in the loop” models towards the symbiosis of human-AI intelligent caring in healthcare design, resourcing, evaluation, and improvement (2). This new values-based approach to technology development we propose (2) reinforces the values established in professional practice through technologies themselves. For example, new technologies are being developed with Artificial Compassion design methods and tools (3). The idea, first developed by co-editor and AI technologist Cindy Mason (4) takes the science and wisdom of human compassion and embeds it into technologies and algorithms to enhance human lives.

The present innovative digital health landscape creates an opportunity to rapidly advance human-AI intelligent caring through enhanced educational curricula and transformative learning experiences (2, Figure 2).
